# Closing the loop of deep brain stimulation

**DOI:** 10.3389/fnsys.2013.00112

**Published:** 2013-12-20

**Authors:** Romain Carron, Antoine Chaillet, Anton Filipchuk, William Pasillas-Lépine, Constance Hammond

**Affiliations:** ^1^Aix Marseille Université UMR 901Marseille, France; ^2^Institut national de la Recherche Médicale et de la Santé Inserm, INMED UMR 901Marseille, France; ^3^APHM, Hopital de la Timone, Service de Neurochirurgie Fonctionnelle et StereotaxiqueMarseille, France; ^4^Laboratoire des Signaux et Systèmes(L2S), CNRS UMR 8506Gif-sur-Yvette, France; ^5^Université Paris Sud 11, UMR 8506, SupélecGif-sur-Yvette, France; ^6^Centre national de la recherche scientifiqueParis, France

**Keywords:** DBS, mechanisms, antidromic, open loop, closed loop

## Abstract

High-frequency deep brain stimulation is used to treat a wide range of brain disorders, like Parkinson's disease. The stimulated networks usually share common electrophysiological signatures, including hyperactivity and/or dysrhythmia. From a clinical perspective, HFS is expected to alleviate clinical signs without generating adverse effects. Here, we consider whether the classical open-loop HFS fulfills these criteria and outline current experimental or theoretical research on the different types of closed-loop DBS that could provide better clinical outcomes. In the first part of the review, the two routes followed by HFS-evoked axonal spikes are explored. In one direction, orthodromic spikes functionally de-afferent the stimulated nucleus from its downstream target networks. In the opposite direction, antidromic spikes prevent this nucleus from being influenced by its afferent networks. As a result, the pathological synchronized activity no longer propagates from the cortical networks to the stimulated nucleus. The overall result can be described as a reversible functional de-afferentation of the stimulated nucleus from its upstream and downstream nuclei. In the second part of the review, the latest advances in closed-loop DBS are considered. Some of the proposed approaches are based on mathematical models, which emphasize different aspects of the parkinsonian basal ganglia: excessive synchronization, abnormal firing-rate rhythms, and a deficient thalamo-cortical relay. The stimulation strategies are classified depending on the control-theory techniques on which they are based: adaptive and on-demand stimulation schemes, delayed and multi-site approaches, stimulations based on proportional and/or derivative control actions, optimal control strategies. Some of these strategies have been validated experimentally, but there is still a large reservoir of theoretical work that may point to ways of improving practical treatment.

## Introduction

Continuous high-frequency deep brain stimulation (open-loop DBS referred to as HF DBS or HFS) is a widely used therapy, particularly to treat movement disorders such as essential tremor (Benabid et al., 1991, 1993; Schuurman et al., 2000), Parkinson's disease (Benabid et al., 1987, 2009; Limousin et al., 1998; Krack et al., 2003; Deuschl et al., 2006; Castrioto et al., 2011), and generalized dystonia (Coubes et al., 2000; Vidailhet et al., 2007; Isaias et al., 2009). The HF DBS procedure consists in implanting a multi-contact lead, typically in either the ventral thalamus, the internal segment of the globus pallidus (GPi) or the subthalamic nucleus (STN), depending on the pathology (Follett et al., 2010; Moro et al., 2010), and applying short-duration stimulating pulses (60–400 μs) at a constant high frequency (approximately 130 Hz). Several parameters, including mode of stimulation (monopolar vs. bipolar), electrode polarity (which contact of the quadripolar lead is negative), pulse width and intensity of stimulation, are determined for each patient by a highly trained clinician. The aim of this adjustment is to optimize motor improvement while minimizing any side effects. The initial programming can take up to 6 months before optimal results are obtained (Volkmann et al., 2006; Bronstein et al., 2011).

HFS has also been tested in several psychiatric diseases such as obsessive compulsive disorder (OCD), in several different anatomical targets (ventral limb of internal capsule VLIC, nucleus accumbens NAc or limbic STN) (Nuttin et al., 1999; Mallet et al., 2008), in Gilles de la Tourette syndrome (CM-Pf nucleus of the thalamus, anteromedial GPi, VLIM/NAc, Vop (Visser-Vandewalle, 2007) or in refractory depression (subgenual cortex: CG25) (Mayberg et al., 2005; Holtzheimer and Mayberg, 2011). HFS has been extended to other target brain nuclei or fiber tracts for the treatment of several other pharmaco-resistant brain pathologies. For the treatment of trigemino dysautonomic headaches such as refractory cluster headaches, HFS applied in the region of the posterior hypothalamus (Matharu and Zrinzo, 2010) shows positive preliminary results (Franzini et al., 2003; Leone et al., 2006). For obesity, a potential therapeutic role of HFS in the lateral hypothalamus is being investigated (Quaade et al., 1974; Halpern et al., 2011; Torres et al., 2011; Melega et al., 2012). The SANTE trial (Electrical stimulation of the anterior nucleus of thalamus) for refractory epilepsy (Fisher et al., 2010) gave promising results. Recently, HFS of the fornix/hypothalamus in Alzheimer's disease was used to stimulate the memory circuits (entorhinal cortex, hippocampus) and is linked to improvements at 1 year follow-up (Laxton et al., 2010; Smith et al., 2012).

The main sites for HFS are located along the various (motor, associative, limbic) thalamo-cortical loops and in hypothalamic regions. These sites have in common the presence of pathological activities (dysrhythmia and/or hyperactivity) as shown by recordings in patients both at the single neuron level (via microelectrode probes during surgery) and at the population level (via implanted DBS leads or imaging studies). There are examples of pathological activity in rhythmic oscillations and pairwise synchrony in the ventral thalamus Vim/Vop of patients suffering from essential tremor (Hanson et al., 2012); augmented synchrony of neuronal firing, loss of specificity of the receptive fields, and increased firing rates with bursts in the STN and GPi of Parkinson's disease (PD) patients (Hutchison et al., 1998); rhythmic oscillations in the Vop of patients suffering from Tourette syndrome (Marceglia et al., 2010); high-frequency discharge with bursting in the limbic STN (Welter et al., 2011) or in the ventral caudate nucleus of patients showing OCD episodes during surgery (Guehl et al., 2008); increased activation of regional cerebral blood flow that may be reversed by several anti-depressant therapies in the subcallosal cingulate gyrus (SCG) in patients with depression (Hamani et al., 2012) and in areas specifically activated in patients experiencing acute cluster headaches but not in other causes of head pain (May et al., 1998).

This review aims at examining why it has been so difficult to discern the mechanisms underlying the HFS-mediated clinical improvements. In particular, it underlines the role played by the combined antidromic and orthodromic effects of HFS (Part I). At the same time, recent theoretical developments and empirical findings are surveyed, revealing how DBS can now be diversified to optimize its benefits especially by exploiting models and/or measurements on the brain structures involved (Part II).

### Part I: open-loop HFS evokes axonal spikes that follow two routes, antidromic and orthodromic

#### The hypothesis of the local inhibitory action of HFS

Considerable effort has been directed toward understanding the mechanisms behind HFS-driven clinical improvement. HFS was first thought to mediate its clinical benefit by local inhibition of the stimulated neurons. This hypothesis was raised partly because the structures targeted for HFS are exactly those previously targeted for a lesion, and show hyperactivity and dysrhythmia. But similar consequences do not imply similar causes: similar behavioral effects between the lesion of a nucleus, its inhibition by pharmacological agents or its high-frequency stimulation do not allow us to conclude that HFS has an inhibitory action (Aziz et al., 1991; Schuurman et al., 2000). Since intracellular recordings at the site of stimulation during HFS also produced contradictory results (Magarinos-Ascone et al., 2002; Garcia et al., 2003; Tai et al., 2003; Meissner et al., 2005), this question is still under debate (Table 1). Nevertheless, the effect of HFS on neuronal activity in structures connected to the stimulated neurons seems to hold the key.

**Table 1:**
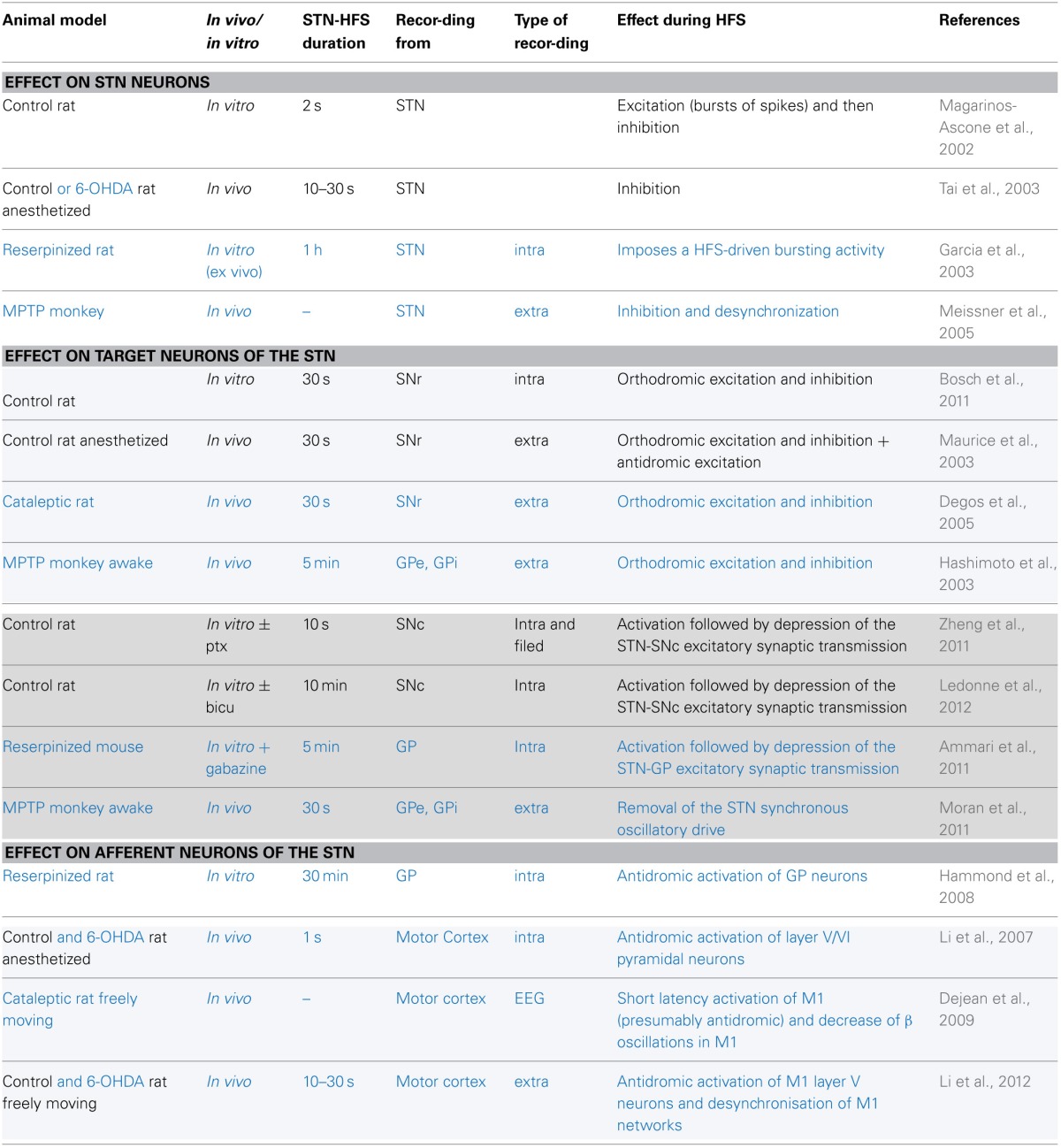
**Electrophysiological effects recorded during STN-HFS in control animals (in black) or in animal models of Parkinson's disease (in blue)**.

Experiments are grouped on the basis of the results obtained. The different gray shadings indicate the different groups. Bicuculline (bicu), gabazine, or picrotoxin (ptx) were sometimes added to the extracellular medium to block GABAA receptor-mediated transmission and to record in isolation STN-SNr or STN-SNc or STN-GP glutamatergic synaptic transmission.

#### HFS has distal effects via HFS-evoked axonal spikes

Recordings far from the stimulating site, in the target regions, where stimulation artifacts are less of a problem, give interesting results. There is a general consensus on the fact that HFS induces distal effects via propagation of evoked spikes along axons. Obviously, however, several factors can determine the nature and strength of this axonal activation.

**Figure 1:**
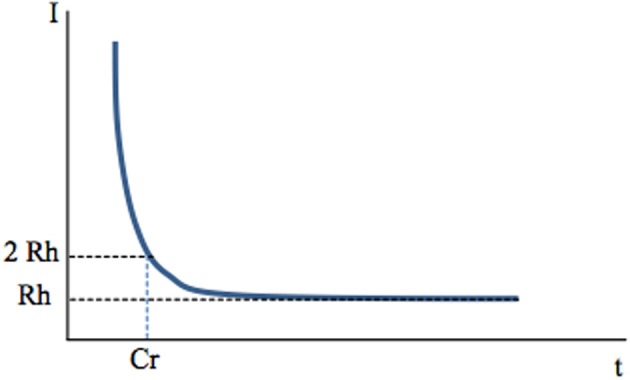
**Chronaxy**. Deep-brain high-frequency stimulation is applied through an extracellular stimulating electrode. Stimulation usually consists of 60–400 μs pulses applied at a frequency of 100–130 Hz. It activates one or several neuronal elements close to the stimulating electrode: somato-dendritic trees, axons, axon terminals. This depends on stimulation parameters, since each of these elements has a specific chronaxy (Ranck, 1975) which determines whether they are activated by a given stimulation. *A single, short-duration, extracellular stimulating pulse preferentially activates axons* The link between the intensity of stimulation (Y axis) and the minimal duration of this stimulation (pulse width, X axis) needed to activate a given element (muscle fiber or neuron, soma or axon of a neuron) is hyperbolic and described by the Weiss law: *I* = Rh (Cr/t +1). When the current intensity of a pulse is decreased, its duration (pulse width) must be increased to produce constant effects i.e., activate the given neuronal element. The asymptote to the X axis defines the *rheobase (Rh)*. It corresponds to the minimal current intensity needed to activate an element (muscular or neuronal). If applied at an intensity lower than rheobase, the stimulus will never activate a given element, whatever its duration (*t*). The minimum duration required for a constant electric current of twice the rheobase to excite tissue is the *Chronaxy (Cr)*. When *I* = 2Rh, t = Cr The concepts of “chronaxie” and “rheobase” were introduced in 1909 by the French physiologist L. Lapicque. The root word “rheo” means current and the root word “chron” means time. The chronaxy is used to quantify the excitability of an element. The element is more excitable when its chronaxy is short. Chronaxies of the different elements of the nervous system differ by a factor of 5 to 300. Large myelinated axons of the central nervous system have a chronaxy of 30–300 μs and around 500 μs for non-myelinated axons, whereas that of somas and dendrites is around 1–10 ms (Ranck, 1975). In the cat visual cortex, Nowak and Bullier (1998) found similar results with a chronaxy of around 270 μs for axons of the subcortical white matter and of 15 ms for somas in the cortex. Therefore, a stimulating pulse of 60–400 μs duration preferentially activates axons. Larger axons have a lower threshold of activation because the intracellular resistance to longitudinal ionic flux is low as a result of the higher percentage of ions that carry the current per length unit. Therefore, for a given current applied, the large axons are those most easily depolarized.

A first factor may be the frequency of stimulation. If short-duration (60–400 μs) stimuli applied at 0.1–1 Hz via an extracellular electrode activate axons (Figure 1), would the same stimuli applied at high frequency (100–130 Hz) also preferentially activate axons? Holsheimer measured chronaxy and rheobase during 130 Hz stimulation of the ventral intermediate nucleus of the thalamus (Vim) or internal pallidum (GPi) in the context of essential tremor or PD tremor, respectively (Holsheimer et al., 2000). He determined the pulse duration needed to stop tremor at threshold intensity using the Weiss method. The mean values were 65 ± 26 μ s for Vim and 75 ± 25 μ s for GPi. He concluded that the elements stimulated under the clinical parameters (60 μs, 130 Hz, 2.5–3.0 V) were large-diameter axons whose chronaxies are between 30 and 300 μ s.

Another variable is the distance between the DBS electrode and neuronal elements. Ranck proposed that high-intensity monopolar cathodic pulses silence proximal neural elements and weakly activate more distal ones, thus, delineating a shell where neural elements are clearly activated (Ranck, 1975). (Gustafsson and Jankowska, 1976) confirmed this hypothesis in cat motoneurons. They recorded the response of a given motoneuron to a stimulus applied via an extracellular electrode positioned at different distances from the recorded motoneuron. A response was considered as direct (not through a network) when the evoked action potential had a latency shorter than 5 ms. Action potentials were evoked with the lowest intensity when the stimulating electrode was close to the axon initial segment, and with a higher intensity when it was close to the dendritic arbor.

The volume of neuronal tissue stimulated depends on the electrode's characteristics. Most neurosurgical teams use the same DBS quadripolar lead with 4 contacts 1.5 mm high and either 0.5 mm or 1.5 mm apart (Model 3389 vs. 3387, Medtronic®, Minneapolis). Models indicate that a monopolar cathodic stimulation activates axons in a radius of 2.5 mm around the negative plot (Wu et al., 2001; McIntyre et al., 2004; Hemm et al., 2005). Coubes's team recently modeled the volume of tissue stimulated (homogenous and isotropic model) as a function of the geometrical characteristics of the electrode contacts (Vasques et al., 2010).

In conclusion, HFS evokes axonal spikes which propagate along axons in orthodromic and antidromic directions. Orthodromic spikes propagate toward axon terminals, where they may evoke transmitter release and postsynaptic potentials in target neurons. Antidromic spikes propagate in the reverse direction toward afferent networks, where they may have complex effects. We will separately analyze the respective effects of HFS-evoked orthodromic and antidromic axonal spikes.

#### HFS functionally de-afferents the stimulated nucleus from its downstream target networks

The fact that extracellularly-applied HFS activates axons inside or near the stimulated site and generates axonal spikes that orthodromically propagate to axon terminals is attested by the increased or decreased activity recorded from target neurons of the stimulated structure in animal models of PD *in vivo* (6-OHDA rats, MPTP-treated monkeys) or in patients (Perlmutter et al., 2002; Anderson et al., 2003; Hashimoto et al., 2003; Maurice et al., 2003; Bosch et al., 2011; Walker et al., 2011). Since the duration of stimulation used in these studies is relatively short (ranging from several milliseconds to minutes) compared with the clinical duration of stimulation (lifetime), it is impossible to know whether this excitatory or inhibitory effect is transient (minutes, hours) or persistent (days). Yet the hypothesis of persistent activation of target neurons of the STN during STN-HFS (>100 Hz) cannot be readily reconciled with the classical model of Alexander (Alexander et al., 1986, 1990), nor with clinical observations such as the lack of interference of thalamic HF DBS on motor control (Takahashi et al., 1998; Flament et al., 2002; Suilleabhain et al., 2003). In that case, activation of GABAergic output neurons of the basal ganglia (SNr/GPi) by STN stimulation would aggravate akinesia by reinforcing the inhibitory tonus on thalamic neurons.

**Figure 2:**
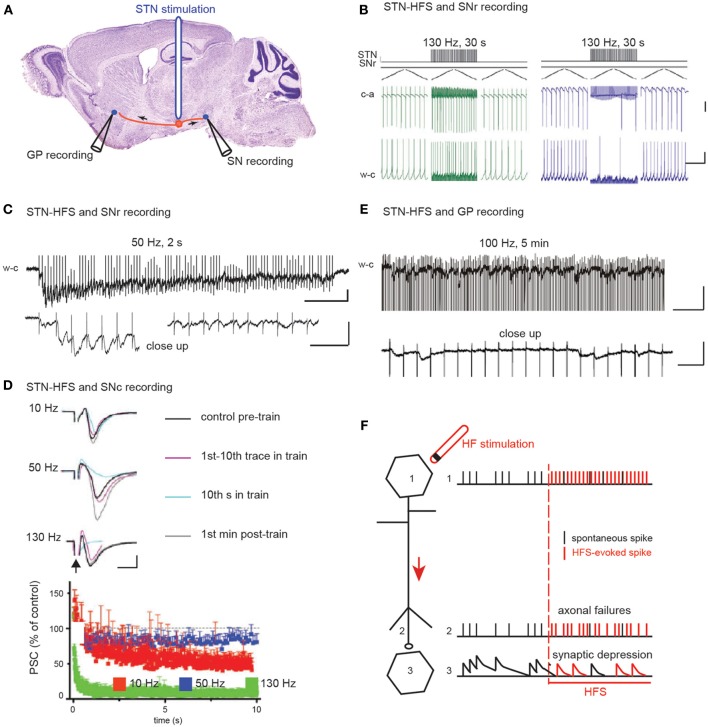
**The two hypotheses on the effect of STN HFS-evoked orthodromic spikes on the activity of substantia nigra and pallidal neurons. (A)** Schematic illustration of the experimental design showing the stimulation and recording sites. **(B)** HFS-evoked orthodromic spikes in STN axons evoke excitatory (left) and inhibitory (right) responses in SNr neurons recorded in cell-attached (c-a) or whole-cell (w-c) configuration in voltage (top) or current (bottom) clamp mode. Scale bars: 100 pA top, 5 mV bottom, 400 ms. Adapted from Bosch et al. (2011). **(C–E)** HFS-evoked orthodromic spikes in STN axons evoke low amplitude EPSCs in SNr **(C)**, SNc **(D),** and GPe **(E)** neurons. Bottom traces in **(C)** and **(E)** are close ups to the top traces at the beginning **(C)** left and at the end **(C)** right, **(E)** of the stimulation. Scale bars are 50 pA, 200 and 20 ms in **(C)**, 50 pA and 5 ms in **(D)** and 50 pA, 200 and 20 ms in **(E)**. **(C)** from Ammari and Hammond (personal communication), **(D)** adapted from Zheng et al. (2011) and **(E)** adapted from Ammari et al. (2011). **(F)** Schematic illustration of the possible mechanisms underlying HFS-induced depression of synaptic transmission.

Several studies on STN-HFS (Figure 2A) have proposed that HFS de-afferents the stimulated nucleus from its target neurons. Two different de-afferentation mechanisms have been proposed for a common consequence: the regularization of target neurons activity. The first mechanism proposed (Hashimoto et al., 2003; Maurice et al., 2003; Degos et al., 2005; Bosch et al., 2011) is that STN-HFS (100 μs pulses at 130 Hz during 30 s) imposes a concomitant synaptic excitation-inhibition on substantia nigra reticulata (SNr) or globus pallidus (GP) target neurons, which tightly regulates SNr or GP activity. This arises in the SNr from the concomitant activation of glutamatergic subthalamo-nigral and GABAergic pallido-nigral fibers (the latter pass through the rat and non-human primate STN) (Parent and De Bellefeuille, 1983; Sato et al., 2000; François et al., 2004) (Figure 2B). This balance seems to favor excitation in 6-hydroxydopamine-treated rats. At a higher frequency (180 Hz), STN-HFS no longer evokes significant inhibitory or excitatory responses.

The second mechanism proposed is that prolonged STN-HFS depresses glutamatergic synaptic transmission between subthalamic terminals and target neurons (Figures 2C–F). Indeed, EPSCs evoked in SNr neurons by STN stimulation in the continuous presence of blocker of GABA_A_ receptor-mediated transmission rapidly decrease in frequency and amplitude during STN-HFS at 50 Hz in the basal ganglia slice of control mice (Figure 2C). Accordingly, in rodent brain slices, STN-HFS disrupts synaptic excitation onto target SNr or SNc neurons (Zheng et al., 2011; Ledonne et al., 2012). EPSCs follow the first few stimuli before their amplitude rapidly declines (Figures 2C,D). STN-HFS induces a rapid and input-specific suppression of the synaptic transmission from STN to SN neurons that is maintained throughout the stimulation. Finally, in the basal ganglia slice from reserpinized mice, the spontaneous, large-amplitude, sometimes recurrent, complex EPSCs evoked by single stimuli in the STN disappear in GP neurons during STN-HFS applied for several minutes (100 μs, 100 Hz) and are replaced by very low-amplitude, regularly-spaced EPSCs at around 20 Hz (Ammari et al., 2011) (Figure 2E). This reduction of the STN-pallidal synaptic efficacy is also reported in the awake MPTP-treated monkey with multielectrode recordings in the GP during STN-HFS (Moran et al., 2011).

Similar observations have been obtained in other networks. In a pioneering imaging study in thalamo-cortical slices using a voltage-sensitive dye, excitation of cortical neurons by stimulation of the ventrolateral thalamus does not follow HFS above 80 Hz (Urbano et al., 2002). Subcortical fiber stimulation (90 μs, 125 Hz during 30 s) to mimic thalamic HF DBS for tremor (stimulation of VL thalamus fibers projecting to M1), induces an initial transient, AMPA/NMDA mediated depolarization or inward current in primary motor cortex (M1) neurons (layers II/III, V, and VI) followed by a depression. When the size of the 20th or 100th EPSCs is measured during the HFS train, a marked failure/depression of transmission is present at frequencies above 50 Hz (Anderson et al., 2003). Imaging of the subcortical white matter during HF DBS of the subgenual cingulate (for depression) shows the reduced activation of cortical projection sites (Mayberg et al., 2005).

The mechanism underlying the decline of synaptic transmission during HFS includes presynaptic inhibition of glutamate release via adenosine A1 receptors (Bekar et al., 2008) but not via metabotropic GABA or glutamate receptors (Zheng et al., 2011). It also includes axonal transmission failure (Figure 2F), which halts synaptic transmission, since STN-HFS at 50 and 130 Hz, respectively, strongly diminishes or abrogates afferent volley responses extracellularly recorded in the SNc (Zheng et al., 2011). Elevated extracellular K^+^ due to HFS might in fact impair action potential conduction (Bellinger et al., 2008; Zheng et al., 2011).

In conclusion, STN-HFS evokes axonal spikes that orthodromically propagate and depress glutamatergic synaptic transmission to postsynaptic neurons (Figure 2F, Table 1). As a result, the pathological electrophysiological activity of glutamatergic STN neurons no longer propagates to its target neurons. Instead, HFS injects a continued, low amplitude, postsynaptic noise, thus, imposing a new resting state in the network. HFS of glutamatergic neurons/axons prevents these neurons from influencing the activity of target neurons. This can be described as a reversible “functional de-afferentation” of downstream target neurons from the stimulated nucleus.

Nevertheless, the functional de-afferentation of nuclei downstream of the stimulated STN does not explain alone the beneficial clinical effects of HFS. An optogenetic study (Gradinaru et al., 2009) using light-sensitive neuronal modulators driven by cell type-specific promoters showed that increasing or decreasing activity of glutamatergic excitatory STN neurons is not sufficient to mimic the beneficial effect of STN-HFS in 6-OHDA-treated rats.

#### HFS of a nucleus prevents it from being influenced by its afferent networks

**Figure 3:**
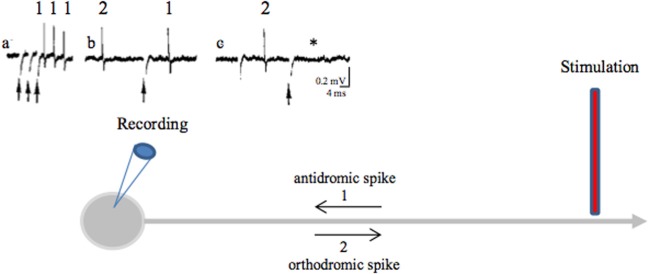
**Antidromic spikes**. The antidromic propagation of a spike refers to its conduction in a direction opposite from the normal (orthodromic) direction (away from axon terminals to soma instead of propagating from the initial segment of the axon, close to the soma, toward axon terminals). To evoke antidromic spikes, axons are directly stimulated with a suprathreshold stimulus. Evoked spikes propagate in both directions (orthodromic and antidromic). This shows that axons do not have a preferential direction of conduction: the direction of propagation is given by the synapses, which are unidirectional [from the axon terminal (presynaptic element) to the postsynaptic element]. Antidromic activation is often used in a laboratory setting to confirm that a recorded neuron projects to the structure of interest. During HF DBS, antidromic spikes are evoked because an extracellular stimulation preferentially activates axons (axon terminals, passing axons). *Criteria for identification of an antidromic spike are:* (i) Stability of latency (because there are no synapses between the stimulating and recording sites), (ii) Faithful responses to high rates of stimulation (for the same reason as above), (iii) Collision of the antidromic spike (1) with an orthodromically traveling spike (2) because they meet along the same axon and annihilate each other. As antidromically activated units sometimes do not fire spontaneously, in order to perform a collision test the action potentials are orthodromically evoked by another stimulation or by depolarizing the soma with the recording electrode. (a) Three spikes recorded from the soma (blue recording electrode) in response to three stimuli (arrows) applied at the axon (red stimulating electrode) (three superimposed traces). (b) A spontaneous orthodromic spike (2) does not suppress the evoked spike (1) when it is recorded long before the stimulation but does so (c, ^*^) when it is recorded 10 ms before the stimulation. These results show that spike 1 is an antidromic spike: it has a fixed latency (a), it faithfully follows high frequency stimulation (a) and it collides with spontaneous spikes (c).

**Figure 4:**
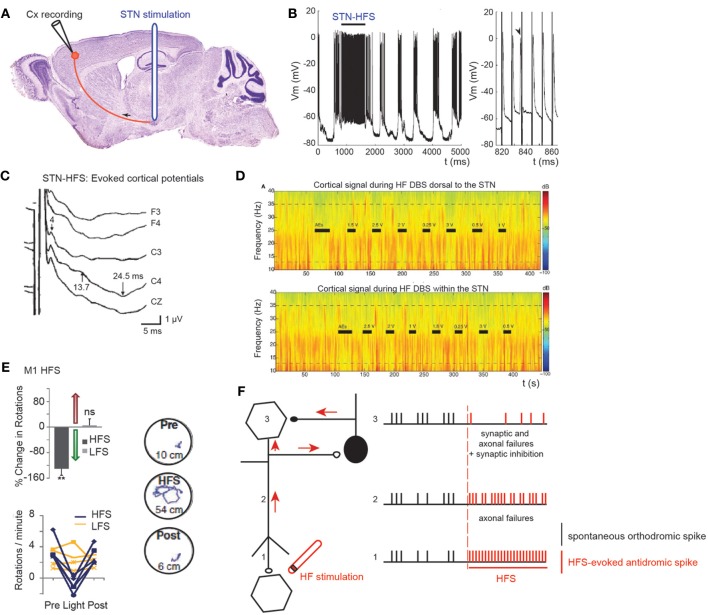
**Effect of STN HFS-evoked antidromic spikes on cortical activity. (A)** Schematic illustration of the experimental design showing the stimulation and recording sites. **(B)** Antidromic spiking in cortical neurons evoked by STN-HFS (intracellular recording in the motor cortex). Black bar indicates the period of STN-HFS. Stimulation artefacts are removed. Right trace is a close-up of **(B)** left. Arrow shows a spontaneous spike before stimulation with subsequent loss of an antidromic spike caused by collision. Adapted from Li et al. (2007). **(C)** Evoked potentials in the ipsilateral motor cortex in response to bipolar stimulation of the dorsal STN. Latencies of the peaks were 4, 13.7, and 24.5 ms. The short-latency negative-evoked potential has a peak latency of 4 ms in bilateral frontal and central leads (C3, C4, Cz). Adapted from Kuriakose et al. (2010). **(D)** STN-HF DBS induces beta attenuation in motor cortex. Spectrogram of single cortical ECoG channel during HF DBS (top) 1.5 mM dorsal to the dorsal border of the STN and (bottom) within the STN (2.6 mM below dorsal border) from a representative patient. Black bars indicate the full time that HF DBS is on. The bars marked “AEs” indicate the period when HF DBS is increased from 0 to 3 V to test for adverse clinical effects; these segments were not used in analyses. The color scale indicates the level of log beta power on a decibel scale. Note the power rebound when STN DBS is turned off. Adapted from Whitmer et al. (2012). **(E)** From top to bottom and left to right. Optical HFS (130 Hz, 5-ms pulse width) reduces amphetamine-induced ipsilateral rotations in 6-OHDA Thy1::ChR2 mice (*P* < 0.01, *n* = five mice) in contrast to optical LFS (20 Hz, 5-ms pulse width, *P* > 0.05, *n* = four mice); *t*-test with *m* = 0. Sample paths before, during and after HFS are shown (100 s each, path lengths in cm). Adapted from Gradinaru et al. (2009). **(F)** Schematic illustration of the possible mechanisms underlying the antidromic effects of HFS. Spontaneous orthodromic spikes are in black and HFS-evoked antidromic spikes in red.

A stimulation applied in a nucleus also evokes axonal spikes that antidromically propagate along afferent axons and in axons passing through or near the stimulated site. Antidromic propagation refers to the propagation of axonal spikes from their point of initiation close to the stimulating electrode toward somas, i.e., in the opposite direction to physiological spikes, which propagate in the orthodromic direction toward axon terminals. Experimenters differentiate antidromic from orthodromic responses by the stable latency of antidromic spikes (there are no synapses between the stimulating and recording points), their collision with spontaneous orthodromic spikes and their ability to follow high frequency stimulation (Figure 3). Antidromic spikes do not reliably invade somas and activate at best a subset of afferent neurons. This depends on the diameter and myelination of the stimulated axons as well as on the geometric ratio between axon and soma diameters. Antidromic spikes may also propagate in recurrent axonal collaterals on their way to somas, activating synaptic transmission that impinges onto other projection neurons or local interneurons. At this point they behave like orthodromic spikes in a network. The overall result depends on the balance between the antidromic invasion of somas and the propagation of antidromic spikes in axonal branches and on how these spikes evoke synaptic responses in the long term (see Figure 4F).

STN-HFS may have widespread antidromic effects because numerous fibers are present inside and around the STN (Mathai et al., 2013). A radius of 2.5 mm around the negative plot, usually at the dorsal border of the STN, contains cortico-subthalamic and pallido-thalamic axons of Forel fields (lenticular ansa and lenticular fasciculus), subthalamo-nigral and subthalamo-pedunculopontine axons and cortico-spinal axons running in the internal capsule. STN-HFS evokes antidromic responses in a subset of pallidal and cortical neurons that directly project to the STN (Kunzle, 1978; Parent and Hazrati, 1995). Antidromic spikes are clearly recorded in the GP during STN-HFS (Hammond et al., 2008). If these antidromic spikes also propagate in the complex network of local GABAergic collaterals that synapse onto other GP neurons (Sims et al., 2008), this may have strong consequences on the activity of subpopulations of GP neurons.

Short-latency antidromic activation of M1 neurons is recorded during STN-HFS in anaesthetized or freely moving rats *in vivo* (S. Li et al., 2007; Dejean et al., 2009) (Figures 4A,B). The frequency of antidromic spikes is higher in dopamine-depleted state (6-OH DA treated animals) (Chomiak and Hu, 2007). The absence of dopamine may change the intrinsic membrane properties of cortical neurons, since the somatic gating of antidromic spikes depends on membrane potential (Chomiak and Hu, 2007). Does STN stimulation also antidromically excite motor cortical neurons in patients? This can be studied via the electrode implanted in the STN and scalp recordings (Cunic et al., 2002; Strafella et al., 2004; Eusebio et al., 2009; Kuriakose et al., 2010). STN stimulation evokes cortical potentials at short (1–8 ms), medium (18–25 ms), and long latencies (more than 50 ms) (Ashby et al., 2001; MacKinnon et al., 2005; Kuriakose et al., 2010; Walker et al., 2012). The early cortical response (mean latency 3 ms) recorded in 80% of the hemispheres tested, and which is maximal in the frontal cortical regions, follows 100 Hz stimulation (Ashby et al., 2001; Baker et al., 2002). It is likely to result from the antidromic activation of the cortico-subthalamic pathway, whereas a response at around 23 ms is likely to result from the orthodromic activation of the basal ganglia-thalamo-cortical network (Figure 4C).

Kuriakose and Coll (Kuriakose et al., 2010) also tested the effects of STN stimulation on cortical excitability at interstimulus intervals (ISIs) corresponding to the short- and medium- latency responses using trans-magnetic stimulation (TMS) in different current directions to determine whether the changes occurred at the cortical or subcortical levels. STN stimulation using contacts that produce clinical benefit increases the excitability of the ipsilateral motor cortex (MEP amplitude) at specific short (2–5 ms) and medium (15–25 ms) latencies tested with TMS in the antero-posterior current direction (activation of cortical pyramidal neurons and interneurons), but not with TMS in the lateral-medial direction (direct activation of corticospinal axons, insensitive to cortical excitability). These sequential increases in excitability might be due to short-latency antidromic activation of cortico-subthalamic projections followed by medium-latency facilitatory basal ganglia-thalamo-cortical interactions following STN stimulation. However, during STN-HFS, the orthodromic polysynaptic responses are likely to disappear (see above), leaving only the antidromic activation of cortical neurons.

Excessive synchronization of neuronal activity in the beta (8–35 Hz) frequency band is one of the main pathophysiological markers of the Parkinsonian state as observed in the widespread sensorimotor network in Parkinsonian animals and in human patients with PD (Brown, 2003; Rosin et al., 2011; Li et al., 2012; Stein and Bar-Gad, 2013). It is currently a matter of debate whether beta hypersynchrony is attenuated during STN-HFS (Rossi et al., 2008; Eusebio et al., 2011, 2012; Whitmer et al., 2012), and to what extent. To answer that question, (Whitmer et al., 2012) positioned subdural cortical surface electrodes over the cortical region from which the hyperdirect cortico-subthalamic pathway originates (using diffusion tensor imaging) and stimulated the STN or its dorsal region. STN-HFS attenuates in a voltage-dependent manner the spectral power in the 5–35 Hz band in the ipsilateral motor cortex (Figure 4D). It also attenuates the coherent oscillations between specific regions of the motor cortex and the STN.

To assess the possibility that antidromic activation plays a significant role in the beneficial effects of HFS, transgenic mice that express light-activated cation channels in afferent fibers to the STN (and not in STN cell bodies) were engineered (Gradinaru et al., 2009). Driving STN afferent fibers with optical HFS robustly and reversibly ameliorates rotational behavior and head position bias in rats made hemiparkinsonian by injection of 6-OHDA unilaterally in the medial forebrain bundle. In contrast, optical low frequency stimulation (LFS, 20 Hz) worsens motor symptoms by increasing ipsilateral rotational behavior. These data strongly suggest that STN HFS ameliorates PD motor symptoms via its antidromic action on M1 network (Figure 4E). Along the same lines, STN-HFS (125 Hz) in freely moving hemiparkinsonian rats evoked antidromic spikes in the ipsilateral (lesioned side) M1 cortex, at frequency that was positively correlated to the therapeutic effect of STN-HFS. Even though their frequency was much lower than the frequency of HFS, antidromic spikes reversed the bursting pattern of ipsilateral M1 cortical neurons (Q. Li et al., 2012).

In conclusion, STN-HFS evokes axonal spikes that antidromically propagate, collide with spontaneous orthodromic spikes and prevent them from influencing the activity of the stimulated nucleus (Figure 4F, Table 1). In the case of the antidromic activation of cortico-subthalamic fibers, antidromic spikes can also excite some cortical interneurons via axon collaterals and depress the activity of pyramidal neurons (Figure 4F). As a result, the pathological synchronized activity no longer propagates from the cortical networks to the stimulated nucleus. This can be described as a reversible “functional de-afferentation” of the stimulated nucleus from its upstream afferent nuclei.

### Part II: latest advances in DBS and perspectives: can closed-loop stimulation lessen the drawbacks of HFS?

Having outlined current understanding of the mechanisms of action of HFS, we now focus on the intrinsic limitations of HFS. We review recent attempts to ameliorate its outcome, especially by exploiting models of the neuronal structures involved and real-time measurements of their activity (closed-loop DBS).

Even excluding problems linked to misplacement of the lead or suboptimal settings of the stimulation parameters, HFS as currently used still has limitations. HFS may be without effect on some PD symptoms or even worsen them, may cause disabling side effects or may become less efficient with time (tolerance/habituation phenomenon) (Hariz et al., 1999). Indeed, STN-HFS does not significantly improve the axial symptoms or cognition impairment that appear with progression of the disease (Castrioto et al., 2011; Rodriguez-Oroz et al., 2012). Whether or not there is any degree of long-term cognitive deterioration clearly ascribable to STN-HFS has not been clearly established yet (Klostermann et al., 2008; Witt et al., 2008; Sáez-Zea et al., 2012). Speech impairment after STN-HFS, however, is common, because of the lack of sustained and global improvement and the worsening of persisting dysarthria (Krack et al., 2003; Pinto et al., 2005; Tripoliti et al., 2011). Similarly, gait and/or balance may significantly deteriorate after Vim-HFS applied for essential tremor (Hwynn et al., 2011). The tolerance effect [habituation of tremor suppression) that appears when setting Vim-HFS for essential or PD tremor also points to important issues regarding the pattern of stimulation (Kumar et al., 2003; Papavassiliou et al., 2004; Barbe et al., 2011). The monomorph continuous pattern of stimulation uselessly increases battery consumption, which strongly impacts the overall cost of the therapy because of the need for periodic internal pulse generator (IPG) replacements. Moreover, the surgical procedure for IPG replacement, though routinely performed, puts the patient at risk of complications, especially of infections. In the end, the trial-and-error selection of the present DBS parameters for each patient is effective because HFS almost immediately impacts PD motor symptoms. However, other therapies utilizing DBS technology may not allow such tuning (for instance, the beneficial effects of stimulation can take weeks to appear in dystonia or obsessive-compulsive disorders). It seems inappropriate to apply the same pattern of stimulation regardless of the state of wakefulness, regardless of whether the patient is at rest or active, lying still or walking, speaking or being silent. Nevertheless, the monomorph type of stimulation has now been offered to patients for over 25 years.

All these limitations are strongly linked to the disproportional signal amplitude and the open-loop nature of the stimulation signal. It therefore, appears to be worth testing other types or modes of delivering the stimulation, to improve the outcome of HFS. The next two sections present recent theoretical or experimental advances in that direction. We distinguish two families of advanced DBS: those that rely on a mathematical model of the dynamics involved, and those that do not. Most of the approaches presented below exploit measurements on cerebral activity (closed-loop stimulation). We start by classifying the latest advances in closed-loop DBS in terms of the nature of the mathematical model they rely on (if any), and then detail the strategy they employ to optimize the treatment.

#### The role of computational models in advanced DBS strategies

Mathematical models recently used to develop closed-loop DBS strategies can be classified into five categories: approaches that require no prior knowledge of the dynamics involved, models describing the phase evolution of the neuronal cells, strategies derived from the Rubin and Terman model, models focusing on the firing rates of the neuronal populations involved and a category covering all other models. Below, we briefly describe these categories before listing the main DBS strategies derived from each of them. A more detailed perspective on the different modeling approaches can be found in a recent survey (Rubin et al., 2012).

***Working without a mathematical model.*** This heuristic approach usually results from medical considerations and is validated or invalidated by *in vivo* or *in vitro* experimental trials. Simplicity is the main reason for relying on non-computational models to generate the stimulation signal. As underlined in Part I, the brain dynamics involved in PD are complex and not yet well understood: the absence of a mathematical model therefore, reduces the uncertainty related to modeling assumptions. It may also involve fewer computational resources, thus, limiting the complexity of the embedded stimulation device, enhancing its reliability and lowering energy consumption.

Several approaches not relying on brain dynamics model have been reported in the literature. Closed-loop, multi-electrode array stimulation of neurons in culture reduces coherent bursting activity (Wagenaar et al., 2005). *In vivo*, in MPTP-treated monkeys, a train of stimuli applied in the GPi each time a spike is detected in the reference structure (primary motor cortex M1) successfully suppresses clinical symptoms (Rosin et al., 2011). Non-invasive closed-loop transcranial alternating current stimulation delivered over the motor cortex at tremor frequency has also been shown to reduce peripheral tremor in PD patients (Brittain et al., 2013).

**Figure 5:**
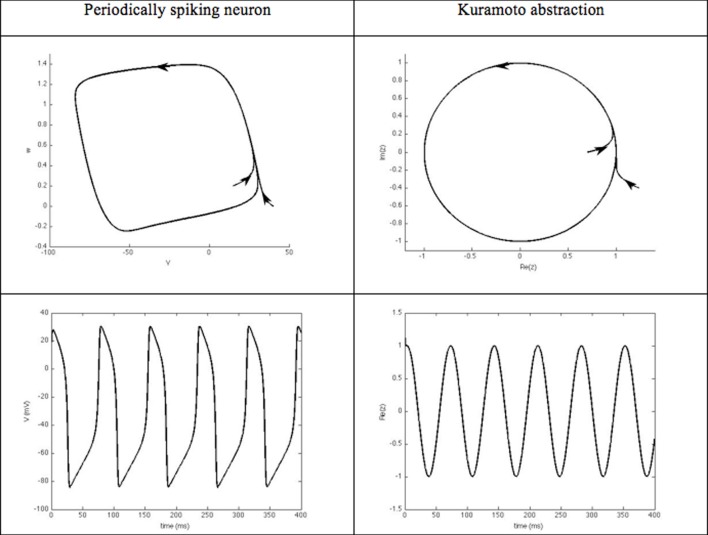
**Kuramato oscillator**. Periodically spiking neurons are characterized by the existence of an attractive limit cycle in their phase portrait. A classical way to reduce the complexity of analyzing their rhythm is to focus on their instantaneous position along this limit-cycle. Using phase-response curves, this abstraction enables the neuron's dynamics to be reduced to a single scalar variable, referred to as its phase. Phase models: For periodically spiking neurons whose limit cycle results from a Hopf bifurcation, the limit cycle can be abstracted to a periodic circle. A normal form of the resulting dynamics is known as the Andronov-Hopf oscillator, which is ruled by the complex equation:
$${\dot{z}}_{i}(t)=\left(j\omega_{i}+1-{\left|z_{i}(t)\right|}^{2}\right)z_{i}(t)+\sum_{i=1}^{N}\kappa_{ij}\left(z_{i}(t)-z_{i}(t)\right),$$
where ω_i_ denotes the natural frequency of the *i*-th oscillator and κ_ij_ are interconnection gains between the *N* oscillators. When the neuronal interconnection keeps the module of *z(t)* constant, the dynamics of the resulting phase θ_i_ takes an even simpler form, known as the Kuramoto oscillator (Kuramoto, 1984):
$${\dot{\theta }}_{i}(t)=\omega_{i}+\sum_{i=1}^{N}\kappa_{ij}\sin\left(\theta_{j}(t)-\theta_{i}(t)\right)$$
Such phase dynamics have been extensively used in the literature to predict synchrony onset in a neuronal population and to derive closed-loop stimulation strategies (Pyragas et al., 2007; Tukhlina et al., 2007; Omel chenko et al., 2008; Franci et al., 2011, 2012).

***Phase models and synchronization.*** These closed-loop DBS approaches focus on the phase dynamics of the neurons of interest. They typically consider periodically spiking neurons. The evolution of their rhythm thus, results from their interaction with other neurons. From a mathematical viewpoint, the dynamics of such neurons is characterized by an attractive limit cycle. The shape of the limit cycle can be quite complicated for physiological models, thus, hampering the mathematical tractability, in particular for large interconnected populations. The instantaneous state of each neuron along this limit cycle is represented by a phase that evolves with time [see (Ermentrout and Terman, 2010; Izhikevich, 2010) for an introduction to the dynamics of periodically spiking neurons and their synchronization properties]. This phase thus, constitutes a simple abstraction of the neuron rhythm, and is well suited to synchronization analysis, which probably explains its success in DBS approaches. When the neuron limit cycle can be likened to a circle, the dynamics governing the phase boils down to the Kuramoto oscillator (Figure 5), which is well documented in the Physics community.

Several works propose innovative closed-loop DBS signals based on this simplified model of phase dynamics. The objectives of each of these contributions can roughly be classified into three categories: the approaches aiming at a desynchronization of the neuronal population (Hauptmann et al., 2005b; Rosenblum et al., 2006; Pyragas et al., 2007; Tukhlina et al., 2007; Danzl et al., 2009; Pfister and Tass, 2010; Franci et al., 2011; Lysyansky et al., 2011), those imposing inhibition (Lysyansky et al., 2011; Franci et al., 2012), and those imposing a prescribed (non-pathological) spatiotemporal pattern on the neuronal population (Liu et al., 2011). Most of these studies are of a theoretical nature and still need experimental validation. A notable exception is (Tass et al., 2012), which provides experimental evidence that phase desynchronization yields not only acute but also long-lasting motor improvements in MPTP monkeys.

**Figure 6:**
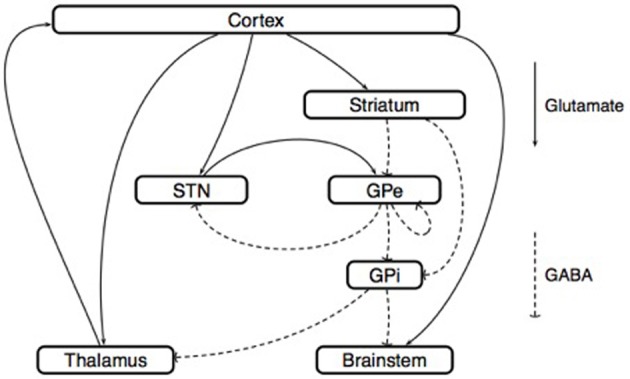
**Model of Rubin and Terman**. This scheme represents the synaptic interconnections, both within the basal ganglia and between their afferent and efferent anatomical structures. This type of circuit representation has generated several basal ganglia models, both at the microscopic and at the mesoscopic scales. Microscopic models: Neural activity is described at the level of each neuron. Following the approach of Hodgkin and Huxley (1952), the dynamics of the membrane voltage *V*_i_(t) associated with the *i*-th neuron is described by a conductance model
$$\begin{array}{l} C_{m}\frac{dV_{i}(t)}{dt}=g\kappa n_{i}^{4}(t)\left(E_{\text{K}}-V_{i}(t)\right)+g_{\text{Na}}m_{i}^{3}(t)h_{i}(t)(E_{\text{Na}}-V_{i}(t))\\ \;+\;g\text{L}(E_{L}-V_{i}(t))+\sum_{j=1}^{n}\kappa_{ij}I_{ij}^{\text{Syn}}(t)+I_{i}^{\text{Ext}}(t) \end{array}$$
where the variables *n*_i_(t), *m*_i_(t), and *h*_i_(t) describe the opening-closing dynamics of different ion channels. In the work (Rubin and Terman, 2004), the parameters that appear in such conductance models are identified for the thalamus, the STN, the GPe, and the GPi. With these parameters in hand, the influence of (open-loop) DBS on model behavior can be analyzed. The conclusion of Rubin and Terman is that the effect of DBS on the STN/GPe/GPi network restores a normal interaction between the cortex and the thalamus, by breaking the pathological patterns generated by the STN/GPe interconnection. Mesoscopic models: Neural activity is described at the level of small neural populations; for example, the region of the sub-thalamic nucleus that is activated by a particular type of movement. Following the approach of Wilson and Cowan (1972), the activity of the *i*-th population is characterized by its firing rate *r*_i_(t), which satisfies the equation 
$$\tau_{i}\frac{dr_{i}(t)}{dt}=-r_{i}(t)+F_{i}\left(\sum_{j\;\in \;E}\kappa_{ij}r_{j}(t-\delta_{ij})-\sum_{j\;\in \;E}\kappa_{ij}r_{j}(t-\delta_{ij})+I_{i}^{\text{Ext}}(t)\right)$$
where *E* and *I* are the set of excitatory and inhibitory populations, respectively. The sigmoid function *F*_i_, called the activation function, characterizes the degree of excitation of the *i*-th population as a function of the inputs that it receives from all the other populations. (Nevado Holgado et al., 2010) used this equation to derive a model of the STN/GPe network, with *E* = {Ctx, STN} and *I* = {Str, GPe}. In this model, the interconnection delays δ_ij_ play a central role in the mechanism that generates pathological beta-band oscillations.

***The model of Rubin and Terman.*** Rubin and Terman introduced a computational model that reproduces some of the phenomena observed in PD and under the effect of HFS (Rubin and Terman, 2004) (Figure 6). In this model, the GPe receives afferent connections from the striatum and the output of the interconnected STN/GPe loop impacts on thalamo-cortical loop activity via the GPi. A fundamental ingredient of their work is a single-neuron model (Terman et al., 2002) that reproduces the behavior of STN, GPe, GPi or thalamo-cortical neurons (different parameters are used for each type of neuron). Then, in each of these regions, several neurons are simulated and interconnected to each other by a synaptic connection model. The whole network can then be simulated in different scenarios (healthy, Parkinsonian and under STN-DBS). In each of these situations, the results obtained from the simulations are coherent with the observations made in the corresponding clinical cases.

Simulations show that during HFS, the rhythmic firing of GPi neurons is replaced by a tonic firing that has a lower impact on the activity of thalamo-cortical relay neurons. This impact is studied using a phase-plane analysis of the dynamics of thalamo-cortical cells. Of course, the question is whether the conclusions of such simulations remain true if the number of neurons, their parameters, or the network interconnections are changed (Pascual et al., 2006). Nevertheless, this theoretical work inspired several authors to develop a suitable control objective for closed-loop DBS including the optimization of the DBS waveform (Feng et al., 2007) and the development of a controllability analysis for a given spatiotemporal pattern of GPi neurons (Liu et al., 2010, 2011).

***Firing-rate models.*** Some studies focus on analyzing the mechanisms underlying pathological oscillations (Gillies et al., 2002; Leblois et al., 2006; Nevado Holgado et al., 2010; Pavlides et al., 2012; Pasillas-Lépine, 2013). They are based on firing-rate models that quantify the state of excitation of different basal ganglia populations, using the formalism introduced by Wilson and Cowan (1972). These models are composed of a small number of ordinary differential equations, for which a stability analysis can be carried out. Interconnection strength and delays may compromise the stability of the network, thus, generating pathological oscillations. There have only been preliminary theoretical studies of the possibility that these models can be exploited for closed-loop DBS purposes (Grant and Lowery, 2013; Pasillas-Lepine et al., 2013). Even though we are not aware of any experimentally-validated closed-loop DBS schemes based on such models, this setting seems well suited to an approach using tools from control theory.

***Stochastic models.*** Neuronal activity is modeled by different types of stochastic process in order to mimic regular, irregular, random and bursting thalamic neurons. Models are exploited to optimize the performance of DBS (Basu et al., 2010; Santaniello et al., 2011; Wilson et al., 2011).

#### Different strategies for closed-loop DBS

In addition to any models they may rely on, advanced DBS strategies may be classified according to the strategy they follow in order to counteract motor symptoms. We next review the prominent strategies that have been followed so far. Table 2 summarizes this comparative analysis.

**Table 2:** **Summarized comparison of closed-loop DBS strategies according to nature of approach, underlying mathematical model (if any), any experimental validation yielded, and overall type of tools (mathematics or simulations) used**.

**Approach**	**Model**	**Experimental validation**	**Analysis tools**	**References**
**ADAPTIVE AND ON-DEMAND**				
On-demand	–	MPTP primates	–	Rosin et al., 2011
On-demand	–	PD patients	–	Graupe et al., 2010
On-demand	–	PD patients	–	Marceglia et al., 2007
On-demand	–	PD patients	–	Little et al., 2013
Adaptive	Conductance-based	–	Artificial neural networks	Leondopulos, 2007
Adaptive	Conductance-based	–	Simulations	Santaniello et al., 2011
Adaptive	Rubin and Terman	–	Optimization	Feng and Fei, 2002
**DELAYED AND MULTI-SITE**				
Delayed and multi-site	Conductance-based	MPTP primates in Tass et al. (2012)	Systems theory in Pfister and Tass (2010)	Hauptmann et al., 2005b
Delayed and multi-site	Phase dynamics	–	Systems theory	Omel chenko et al., 2008
Multi-site	Phase dynamics	–	Simulations	Lysyansky et al., 2011
Delayed	Phase dynamics	–	Systems theory	Rosenblum and Pikovsky, 2004
**PROPORTIONAL, DERIVATIVE, AND INTEGRAL FEEDBACK**				
Proportional and/or multi-site	–	Culture of cortical neurons	–	Wagenaar et al., 2005
Proportional, PID	Phase dynamics	–	Systems theory	Pyragas et al., 2007; Zheng et al., 2011
Nonlinear PID	Rulkov model	–	Systems theory	Tukhlina et al., 2007
Filtered proportional	Hindmarsh-Rose	–	Simulations	Luo et al., 2009
Proportional	Phase dynamics	–	Systems theory	Franci et al., 2011
Filtered proportional	Firing rates dynamics	–	Systems theory	Pasillas-Lepine et al., 2013
**OPTIMAL CONTROL**				
Optimization	Rubin and Terman	–	Optimization	Feng and Fei, 2002
Optimal control	Conductance-based	–	Phase response curve	Danzl et al., 2009

***Adaptive and on-demand stimulation.*** This class of DBS strategies involves automatic tuning of HFS parameters, based on physiological measurements (adaptive DBS), or detection of a pathological situation and activation of the HFS signal in consequence (on-demand DBS). The electrophysiological measurements used to adapt or trigger the stimulation are typically local field potential (LFP) recordings, but can also be single-cell recordings (Rosin et al., 2011) or surface recordings (Graupe et al., 2010). The parameters automatically tuned by these approaches are the frequency, amplitude and cyclic ratio of HFS (Leondopulos, 2007; Santaniello et al., 2011).

The DBS signal can be elaborated based on LFP measurements, after signal processing to reduce noise and remove stimulation artifacts has been performed (Marceglia et al., 2007). A train of stimuli applied in the GPi, 80 ms after the detection of a spike within the primary motor cortex M1 of MPTP primates, efficiently reduces the GPi spike-rate as well as pathological oscillations (Rosin et al., 2011). It also allows stimulation at a much lower frequency (30 Hz instead of 130 Hz). In other cases, LFP recordings from the electrode used for HFS automatically tune HFS parameters using stochastic processes based on experimental data to model regular, irregular, random and bursting thalamus neurons (Santaniello et al., 2011). The automatic adaptation of the stimulation parameters is guided by the objective that the stimulated population follows a prescribed (healthy) reference LFP. In Little et al. (2013), LFP measurements in the STN are made from the stimulation electrode itself. The trigger mechanism that initiates stimulation pulses is tuned to detect changes in beta power that occur on a short time scale (<1 s), raising the possibility that fluctuations in beta power, rather than its average amplitude, are of greatest importance in PD pathophysiology.

All these strategies lead to improvements, both from the physiological and from the energetic point of view. On-demand DBS is beneficial in terms of energy consumption, since the stimulation signal is applied only when needed. Adaptive DBS is also more parsimonious than traditional HFS because the amplitude of the applied signal is usually smaller. From a medical point of view, both on-demand and adaptive strategies are likely to be more physiologically respectful, because the parameters are tuned to improve efficiency, and/or because the stimulation signal is applied less often than for classical HFS. Nonetheless, these strategies continue to exploit the square-shaped, monomorph DBS signals that have proven efficient since the invention of DBS, and do not allow more physiologically-inspired signal shapes to be used. They thus, limit the potential benefit offered by closed-loop DBS.

***Delayed and multi-site stimulation.*** A second family of DBS approaches consists in providing a stimulation signal delayed in time. In most studies, this strategy is coupled with multisite stimulation using several stimulation electrodes. The aim of the delay is to alter synchronization by a superposition of sinusoidal signals that are out of phase and cancel each other out at the measurement location. The measurement (LFP recording) is taken from the stimulated population or from its afferent or efferent structures (Hauptmann et al., 2005a). Several protocols are explored and performances are compared to each other and to standard HFS via numerical simulations. The authors assume that the pathological synchronization is produced by glutamatergic synaptic interactions between STN neurons. Subsequent studies in this direction emphasize either the multi-site (Omel chenko et al., 2008; Lysyansky et al., 2011) or the delay aspects of the problem (Rosenblum et al., 2006; Batista et al., 2010). Multisite stimulation efficiency finds its roots in the synaptic plasticity of the stimulated neuronal population (Pfister and Tass, 2010). This should provide more long-lasting motor symptom reduction, as experimentally shown in MPTP monkeys (Tass et al., 2012). From an experimental point of view, the multi-electrode approach explored with success in Wagenaar et al. (2005) can also be included in this category of control methods.

**Figure 7:**
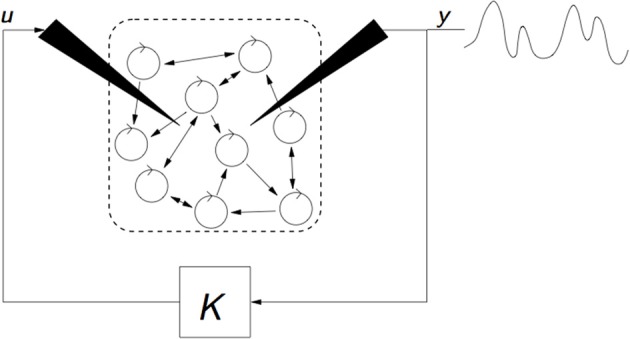
**Illustration of a strategy of closed-loop DBS relying on the measurement y of the mean-field of the targeted neuronal population**. Stimulation input *u* is dynamically established based on measurement *y*, and takes the form *u = G(s)y*, where *G(s)* denotes a filter that can either be proportional (*G(s) = K*) such as in Wagenaar et al. (2005); Leondopulos (2007); Franci et al. (2011, 2012); Liu et al. (2011), include integral or derivative terms such as in Pyragas et al. (2007), or rely on more involved filtering such as in Tukhlina et al. (2007).

***Stimulation based on proportional, integral and derivative control policies.*** This class of closed-loop DBS exploits real-time LFP measurements to elaborate the stimulation signal using tools from control engineering (Figure 7). The stimulation signal is proportional to the recorded LFP or additionally involves dynamic features (such as the LFP integral or derivative). In cultured neurons showing synchronized bursting activity, when the number of available electrodes is limited, the best results are obtained by closed-loop, proportional, firing-rate control (Wagenaar et al., 2005).

**Figure 8:**
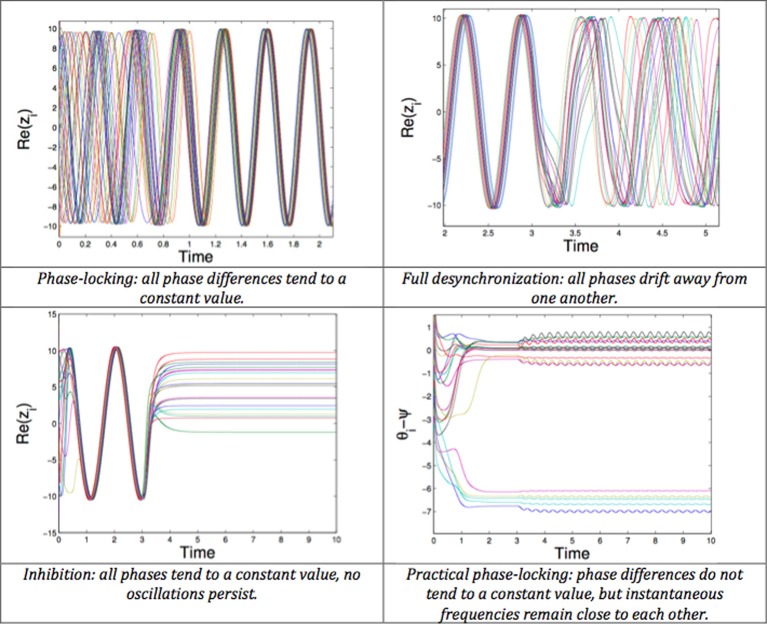
**Possible behaviors of a network of phase oscillators. Top left:** Phase-locking (all phase differences tend to a constant value). **Top right:** Full desynchronization (all phases drift away from one another). **Bottom left:** Inhibition (all phases tend to a constant value, no oscillations persist). **Bottom right:** Practical phase-locking (phase differences do not tend to a constant value, but instantaneous frequencies remain close to each other).

The desynchronizing properties of proportional feedback are also clearly shown using a phase model (Pyragas et al., 2007). They explore two approaches. The first (more theoretical) analyzes the effects of stimulating with a proportional-derivative mean-field feedback, which desynchronizes with an approximation of the system's low order dynamics (first modes of the Fourrier series). The second (based on numerical simulations) considers a more sophisticated model, namely a set of van der Pol oscillators. In this second approach, an integral term is added to the proportional-derivative feedback. Other studies rely on phase models. The mean-field feedback is applied using a particular resonant filter (Tukhlina et al., 2007; Luo et al., 2009) or a purely proportional feedback (without derivative terms) is analyzed with a finite-dimensional model, using an approach based on Lyapunov functions (Franci et al., 2011, 2012). The model of Rubin and Terman is exploited in Leondopulos (2007), Liu et al. (2011). Recent theoretical work (Pasillas-Lepine et al., 2013) suggests that a DBS signal proportional to the recorded STN firing rate is enough to counteract the STN-GPe pacemaker effect in beta oscillations generation, despite possible inherent delays in measurement or stimulation. All these approaches give convincing theoretical results (Figure 8). However, the gap between theoretical analysis and the real neurophysiology of the basal ganglia remains to be explored.

***Optimal control strategies.*** This area of control theory aims at finding a control law for a given system in order to fulfill a certain optimality criterion defined by a cost function involving the state of the system, the control signal or any other relevant variable. The optimal control policy is designed with the aim of minimizing the selected cost function. Optimal control is a very tempting strategy for DBS. The option of including the stimulation signal in the cost function can lead to improvements in terms of energy consumption. The formulation of the control objective as being to minimize a cost function conveniently fits most DBS control objectives (neuronal de-synchronization, inhibition, and beta band oscillation reduction).

The work of Feng et al. (2007) provides a strong basis for the development of optimal control policies for closed-loop DBS. It proposes an optimization procedure to identify efficient DBS waveforms. The proposed cost function accounts for neuronal response correlation (or any measure of pathological criticality), as well as the intensity of the stimulation signal. This cost function is minimized via a genetic algorithm. Even though the selected optimal DBS waveform is then applied in open loop, the proposed cost function is likely to lead to closed-loop developments in the future, by relying on an on-line optimization.

Another approach that exploits optimal control to develop closed-loop DBS strategies is presented in Danzl et al. (2009). The authors propose a strategy to control the spike timing of a neuron by relying on a phase model. The control input is optimal in terms of input energy, and guarantees a charge balance over a stimulation period. It can be used either to impose a prescribed firing pattern or to desynchronize a neuronal population. No such DBS strategies have yet been experimentally validated.

## Conclusion

DBS has now been used for more than 25 years to treat movement disorders. In recent years, its indications have been extended to other clinical areas such as psychiatric diseases, pain or epilepsy. DBS has been empirically performed with a view to inhibiting an overactive or dysrythmic focus, but without a clear understanding of its mechanisms of action. Here we present data showing that DBS exerts distal effects via ortho- and antidromic stimulation of axons, in particular myelinated axons, running close to the stimulated site. The resulting effect is the reduction of abnormal patterns in the afferent and target networks of the stimulated site.

In functional neurosurgery, there is a tendency to ascribe the clinical failures or suboptimal results of DBS to misplacement or suboptimal location of the active contacts of the lead. But better clinical outcomes have also been obtained by experimentally changing the stimulation pattern. Apart from recent developments allowing the so-called interleaving mode to be used, with the application of two concomitant different settings of stimulation, current electronic devices only allow a monomorph square-shaped continuous stimulation to be delivered, regardless of the underlying neuronal activity. Such an invariant stimulation pattern can lead to tolerance and habituation, and thus, loss of DBS efficacy. DBS, whose availability remains limited to a small number of centers in developed countries, is still an expensive therapy. It is bound to be challenged in the future by lesion procedures, which can now be performed non-invasively. Demonstrating DBS superiority over alternative methods may well entail altering the way of delivering the stimulation, with likely subsequent improvements.

Both adaptive and on-demand strategies seem very promising to cost-effectively desynchronize oscillatory pathological patterns as well as delayed multi-site stimulation, though this latter approach requires, by definition, the insertion of several leads and thus, a slightly increased associated surgical risk. These different strategies will have to be evaluated and compared in order to determine the optimal strategy for each specific indication. Theoretical advances through the proportional or dynamic elaboration of DBS signals based on LFP measurements and optimal control strategies are encouraging, but will still require experimental validation.

All the above data plead for a more refined electronic system, incorporating multiple programs of stimulations and feedback information from the target. Open-loop DBS with a square-shaped monomorph pattern should now be regarded as too crude and outdated. There is little doubt that future attempts at closed-loop strategies for DBS will prove helpful and beneficial to patients. Additionally, such strategies will enable us to gather vast quantities of data, shedding light on how DBS works as well as on how certain brain areas function and dysfunction.

### Conflict of interest statement

The authors declare that the research was conducted in the absence of any commercial or financial relationships that could be construed as a potential conflict of interest.
